# Effects of the potential lithium-mimetic, ebselen, on impulsivity and emotional processing

**DOI:** 10.1007/s00213-016-4319-5

**Published:** 2016-06-02

**Authors:** Charles Masaki, Ann L. Sharpley, Charlotte M. Cooper, Beata R. Godlewska, Nisha Singh, Sridhar R. Vasudevan, Catherine J. Harmer, Grant C. Churchill, Trevor Sharp, Robert D. Rogers, Philip J. Cowen

**Affiliations:** Department of Psychiatry, University of Oxford, Warneford Hospital, Oxford, OX3 7JX UK; Department of Pharmacology, University of Oxford, Mansfield Road, Oxford, OX1 3QT UK; Centre for Neuroimaging Studies, PO 089, DeCrespigny Park, London, SE5 8AF UK; School of Psychology, Bangor University, Penrallt Road, Gwynedd, LL57 2AS UK

**Keywords:** Ebselen, Impulsivity, Emotional processing, Lithium-mimetic

## Abstract

**Rationale:**

Lithium remains the most effective treatment for bipolar disorder and also has important effects to lower suicidal behaviour, a property that may be linked to its ability to diminish impulsive, aggressive behaviour. The antioxidant drug, ebselen, has been proposed as a possible lithium-mimetic based on its ability in animals to inhibit inositol monophosphatase (IMPase), an action which it shares with lithium.

**Objectives:**

The aim of the study was to determine whether treatment with ebselen altered emotional processing and diminished measures of risk-taking behaviour.

**Methods:**

We studied 20 healthy participants who were tested on two occasions receiving either ebselen (3600 mg over 24 h) or identical placebo in a double-blind, randomized, cross-over design. Three hours after the final dose of ebselen/placebo, participants completed the Cambridge Gambling Task (CGT) and a task that required the detection of emotional facial expressions (facial emotion recognition task (FERT)).

**Results:**

On the CGT, relative to placebo, ebselen reduced delay aversion while on the FERT, it increased the recognition of positive vs negative facial expressions.

**Conclusions:**

The study suggests that at the dosage used, ebselen can decrease impulsivity and produce a positive bias in emotional processing. These findings have implications for the possible use of ebselen in the disorders characterized by impulsive behaviour and dysphoric mood.

**Electronic supplementary material:**

The online version of this article (doi:10.1007/s00213-016-4319-5) contains supplementary material, which is available to authorized users.

## Introduction

Lithium is best known for its ability to treat acute mania and prevent the recurrence of both mania and depression in patients with bipolar disorder. In addition, lithium is the only psychotropic drug shown reliably to decrease suicidal behaviour (Geddes et al. 2010; Cipriani et al. 2013; Miura et al. 2014), an effect which does not appear to be accounted for solely by its ability to diminish mood disturbance (Cipriani et al. 2013). Consistent with this, a number of studies in non-bipolar patients have shown that lithium decreases the incidence of impulsive aggression (Sheard et al. 1976; Craft et al. 1987; Jones et al. 2011). Such an effect could be important in the management of disorders that have been linked to violent behaviour towards the self and/or others. However, lithium treatment has several drawbacks including poor tolerance, a narrow therapeutic index (resulting in the requirement for blood monitoring) as well as longer-term toxicity, particularly for the kidney (McKnight et al. 2012; Shine et al. 2015). Therefore, a form of drug treatment which has the efficacy of lithium but without its toxicity could be a worthwhile development in the management of impulsive aggression.

Rational design of a lithium-like agent could be pursued based on its mechanism of action, but lithium’s therapeutic target remains unclear. Based on clinically relevant lithium concentrations (0.6–1.2 mM), the two most likely targets are glycogen synthase kinase 3 and inositol monophosphatase (IMPase) (Berridge et al. 1989; Belmaker et al. 1996; Agam et al. 2009). Recently, we reported inhibition of IMPase by ebselen (IC50 1.5 μM), a bioavailable antioxidant drug that has been tested in humans for other diseases including post-stroke neuroprotection and noise-induced hearing loss (Singh et al. 2013; Lynch and Kil 2009; Azad and Tomar 2014).

Using magnetic resonance spectroscopy (MRS), we have previously found that ebselen treatment lowers inositol levels in anterior cingulate cortex in healthy participants (Singh et al. 2015), suggesting inhibition of IMPase by ebselen in humans. In the same investigation, we found that ebselen altered emotional processing by increasing the accuracy of recognition of facial expressions of happiness and disgust in the facial emotion recognition task (FERT). We also found that ebselen decreased learning through reward reinforcement in a probabilistic learning task (Singh et al. 2015). The aim of the present study was to assess further the neuropsychological effects of ebselen by studying the effects of higher dose of ebselen on the FERT and in the Cambridge Gambling Task which assesses reward-seeking behaviour outside of a learning context (Rogers et al. 1999; Clark et al. 2008).

## Methods

### Participants and study design

Ethical approval for the study was obtained from the National Research Ethics Service Committee (NRES), South-Central Oxford B. Twenty healthy participants (7 females, 13 males, mean age 25.1 years, range 20–38 years; mean BMI 22.7 kg/m^2^, range 18.7–30.0 kg/m^2^) were included in the study after giving full informed written consent. Exclusion criteria included a history of any DSM-V Axis I psychiatric disorder (determined using the Standard Clinical Interview for Diagnostic and Statistical Manual for Mental Health Disorders), significant current medical condition, current regular medication (apart from the contraceptive pill), pregnancy or lactation, heavy smoking (defined as more than five cigarettes per day) and having taken part in another study involving an investigational drug within the last 3 months. Participants were asked to maintain stable exercise and diet as well as refrain from alcohol during study participation.

Ebselen capsules and identical matching placebo (containing microcrystalline cellulose) were purchased from Shasun Pharmaceuticals Ltd. Participants were tested twice (7 days apart) receiving on one occasion ebselen and on the other placebo in a randomized, double-blind, cross-over design. Ebselen was administered in 6 × 200 mg capsules in three doses given over 2 days.

On the day before cognitive testing, participants took the first dose at 1 pm and the second dose at 10 pm. The final dose was taken around 3 h prior to cognitive testing. Participants were sent text message reminders a few minutes before they were due to take medication and were asked to confirm receiving the messages. The cognitive tasks were carried out immediately after a magnetic resonance spectroscopy study, the results of which have been reported separately (Masaki et al. 2016).

### Mood, personality and sleep questionnaires

On the screening visit, participants were assessed for baseline depression and anxiety symptoms with the Beck Depression Inventory (Beck et al. 1961) and the state measure of the State-Trait Anxiety Inventory (STAI) (Spielberger et al. 1983), and for personality with the Eysenck Personality Questionnaire (Eysenck and Eysenck 1975). On the morning preceding psychological testing, the Leeds Sleep Evaluation Questionnaire (LSEQ) was completed within 30 min of waking (Parrott and Hindmarch 1980). Before cognitive testing, participants were asked to rate their mood using the Positive and Negative Affective Schedule (PANAS) (Watson et al. 1988), to complete a side effect profile using a 4-point rating scale and also to guess as to whether they had received ebselen or placebo on that randomization arm.

### Cambridge Gambling Task

The Cambridge Gambling Task (CGT) from the Cambridge Neuropsychological Test Automated Battery (CANTAB, version 3.0.0, Cambridge Cognition Ltd., Cambridge, UK) (Rogers et al. 1999; Clark et al. 2008) assesses decision-making and risk-taking behaviour outside a learning context. In each trial, participants are shown ten boxes at the top of the screen, with some boxes being red and some being blue. The ratio of red to blue boxes varies from 9:1, 8:2, 7:3, 6:4, 5:5 and vice versa in a pseudo-random order. Participants are informed that a yellow token is hidden inside one of the boxes and asked to indicate the colour box in which the token is most likely to be hidden, by pressing the colour (RED or BLUE) in a response panel at the bottom of the screen. Following their response, the participants indicate confidence in their selection by betting a proportion of points they are allocated (starting with 100 points). Besides confidence in selection, this measure also assesses the willingness to risk the points they already possess or have accumulated for further real or perceived reward. On each trial, five bets are offered, and each bet represents a fixed percentage of the current total points score (5, 25, 50, 75 and 95 %). Possible bets are presented sequentially in a box on the right of the display and participants touch the box to select the bet. If correct, the bet value is added to their total points on the left of the panel, and if incorrect, it is subtracted from the total points. Participants are asked to accumulate as many points as possible. Following the response, the location of the token is revealed.

Participants perform the task in four blocks of two separate conditions, ascending and descending bet value (the condition order is counterbalanced across participants). In the ascending condition, bets increase at 2.5-s time intervals from 5 to 95 % until participants make their selection. This means that if a participant bets at the first value presented, then they bet only 5 % of their total points, and if they wait for the highest value, they bet 95 % of their total points. In the descending condition, bets start from 95 % and decrease to 5 %. Low bets in the ascend condition and high bets in the descend condition reveal an impulsive betting strategy, while high bets in both conditions reveal a risk-taking or reward-seeking strategy.

From the first stage of the task (selecting the likely colour of the box in which the token is hidden), the outcome measures are deliberation time and quality of decision-making. Deliberation time is the mean latency from the presentation of coloured boxes to participant selection. Quality of decision-making refers to the proportion of trials on which the more likely outcome is chosen. From the gamble stage, the outcome measures are risk taking, risk adjustment and delay aversion. Risk taking refers to the mean proportion of current points that the subject stakes on each gamble when the more likely outcome is selected, and can be regarded as an index of reward seeking or loss aversion. Risk adjustment measures the degree to which a subject varies their risk taking in response to the ratio of red to blue boxes on each trial. Delay aversion is the difference between the risk-taking score in the descend and the ascend condition.

### Facial emotion recognition task

The facial emotion recognition task (FERT) featured six basic emotions—happiness, surprise, sadness, fear, anger and disgust—taken from the Pictures of Affect Series (Ekman and Friesen 1976). These facial expressions had been morphed between each prototype and neutral using techniques described by Young et al. (1997). Morphing involves taking a variable percentage of the shape and texture differences between the two standard images 0 % (neutral) and 100 % (full emotion) in 10 % steps.

Four examples of each emotion at each intensity were presented (from a total set total of ten individuals). Each face was also shown in a neutral expression, giving a total of 250 stimulus presentations. Subjects were asked to assess the facial expression of presented faces as quickly and accurately as possible by pressing one of seven labelled keys. Subjects were informed that facial expression from each category would appear, including neutral, but also that faces would contain different levels of each emotion. Facial stimuli were presented in a random order on a laptop screen for 500 ms then replaced by a blank screen during which time subjects responded. The task was broken down into two parts with an untimed rest period between them. The number of stimuli accurately classified as each emotion, as well as the number incorrectly assigned to each emotion, and reaction times were recorded. The primary outcome measure was the effect of ebselen vs placebo on the accuracy to detect positive (happy + surprise) vs negative (sad + fear + disgust + anger) facial expressions (Post et al. 2015).

### Statistics

Statistical analyses were performed in SPSS version 22. For the CGT, analyses were conducted with repeated-measures ANOVA with ‘treatment’ (ebselen vs placebo) and ‘order’ (placebo first vs ebselen first) as within-subject factors. A similar analysis was carried out for the FERT but ‘emotion’ (positive versus negative facial expression) was added as a further within-subject factor. Significant differences on the ANOVA were followed up with pairwise comparisons using paired samples *t* tests.

## Results

### Subjective state, energy and side effects

At baseline, all participants had low scores on self-rating scales of mood and anxiety (Table S1). There were no main or interactive effects of treatment on mood, assessed using PANAS questionnaire (Table 1). Ebselen was well tolerated and no participants dropped out of the study. Five participants reported feeling drowsy while on ebselen treatment, compared with none reporting this effect while on placebo treatment. Otherwise, there was a low and comparable frequency of side effects reported during ebselen and placebo treatment (Figure S1). There were no significant differences in the subjective measures of sleep assessed using the LSEQ (Figure S2). Participants were more likely to guess correctly that they had received placebo in comparison to ebselen, but the difference was of borderline significance (Table 1).

**Table 1:** Subjective mood ratings using the positive and negative affective schedule (PANAS) questionnaire, during placebo and ebselen study visits. The number of participants who correctly guessed the randomization arm at each visit has also been presented

	Placebo Mean ± SEM	Ebselen Mean ± SEM	Statistical significance
PANAS—positive	28.5 ± 1.6	29.8 ± 1.2	*F*_1, 19_ = 1.7, *p* = 0.204*
PANAS—negative	11.8 ± 0.7	11.9 ± 0.7	*F*_1, 19_ = 0.1, *p* = 0.818*
Correct guesses for randomization	14/20 (70 %)	7/20 (35 %)	*p* = 0.056 (*χ* ^2^)**

*Repeated measures ANOVA; **chi-squared statistic

### Cambridge Gambling Task

**Fig. 1:**
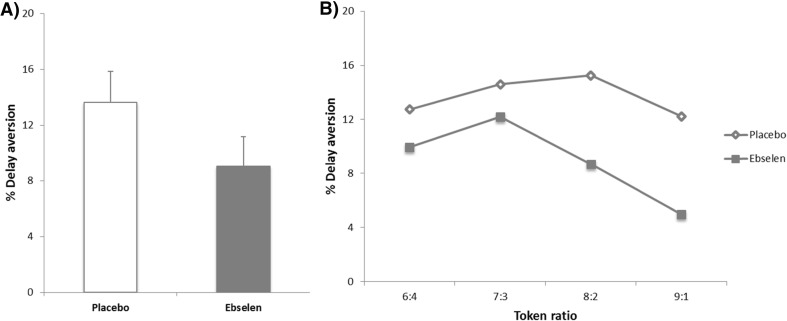
Results of the Cambridge Gambling Task. **a** Ebselen treatment was associated with a significant decrease in the mean delay aversion (main effect of treatment on ANOVA, *F*_1, 18_ = 8.208, *p* = 0.010). **b** The decrease in delay aversion following ebselen treatment was present irrespective of the token ratio presented

There were no significant effects of treatment on the quality of decision-making, deliberation time or risk adjustment (Table 2). Ebselen treatment was associated with a significant decrease in delay aversion (*F*_1, 18_ = 8.21, *p* = 0.010). This effect was present irrespective of the probability of a favourable outcome (ratio of the bets presented) (Fig. 1). Ebselen treatment was also associated with an increase in reward seeking (risk taking) (*F*_1, 18_ = 4.61 *p* = 0.046). There was significant interaction between order and treatment on deliberation time (F_1, 18_ = 38.41, *p* = 0.001). There were no other significant main or interactive effects of order on the remaining measures on the CGT.

**Table 2:** Results of the Cambridge Gambling Task (CGT)

	Placebo Mean ± SEM	Ebselen Mean ± SEM	Statistical significance (repeated-measures ANOVA)
Delay aversion (%)	13.7 ± 2.19	9.0 ± 2.14	*F*_1, 18_ = 8.21, *p* = 0.010
Reward seeking (%)	60.3 ± 1.82	63.9 ± 1.45	*F*_1, 18_ = 4.61, *p* = 0.046
Deliberation time (ms)	1475 ± 99	1408 ± 78	*F*_1, 18_ = 2.50, *p* = 0.131
Quality of decision-making (%)	98.3 ± 0.8	99.3 ± 0.3	*F*_1, 18_ = 0.20, *p* = 0.155
Risk adjustment	2.3 ± 0.15	2.1 ± 0.18	*F*_1, 18_ = 2.88, *p* = 0.107

### Facial emotion recognition task

**Fig. 2:**
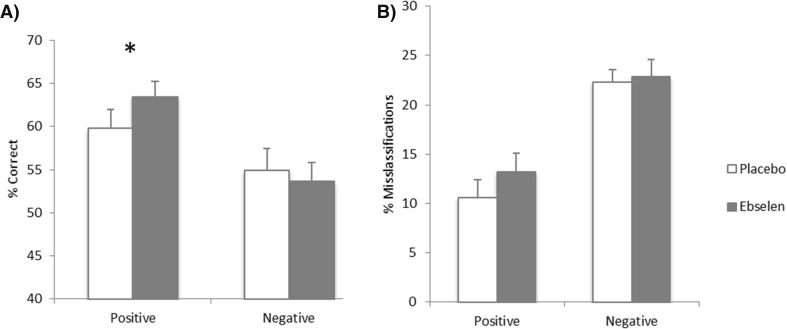
Results of the Facial Emotion Recognition Task. **a** There were significant interactions between treatment and emotion, for accuracy of recognition of positive and negative facial expressions (*F*_1, 18_ = 8.267, *p* = 0.010). Ebselen treatment was associated with a significant increase in the accuracy of recognition of positive expressions without significant effects in recognition of negative expressions. **b** Ebselen treatment was not associated with any significant differences in the misclassifications of emotional expressions. All data are plotted as mean ± standard error of mean, *N* = 20. Values represent the percentage of average total responses for positive (happy + surprise) and negative (angry + disgust + fear + sad) expressions. *p = 0.035

There was a significant interaction between treatment and emotion for the accuracy of recognition of positive vs negative facial expressions (*F*_1, 18_ = 8.27, *p* = 0.010). Follow-up pairwise comparisons revealed that ebselen treatment significantly increased the accuracy of recognition of positive expressions (*p* = 0.035), without significant effects on negative expressions (*p* = 0.38) (Fig. 2). When considering the accuracy of recognition of individual facial expressions, there was a trend towards significant interactions between treatment and emotion (*F*_5, 90_ = 2.15, *p* = 0.067). Follow-up pairwise comparisons revealed that ebselen treatment improved the accuracy of recognition of happy facial expressions (*p* = 0.033) without significant effects on the accuracy of recognition of other emotional expressions (Figure S3, Table S2). Ebselen treatment did not affect the speed to respond to facial expressions or the number of misclassifications (Fig. 2).

There was no significant main effect of order on the accuracy of recognizing positive and negative facial expressions (*F*_1, 18_ = 0.27, *p* = 0.61), but there was a significant interaction between order and treatment when considering accuracy of facial emotion recognition (*F*_1, 18_ = 6.34, *p* = 0.022). However, there was no significant three-way interaction between order, treatment and emotion (*F*_1, 18_ = 0.012, *p* = 0.91).

## Discussion

Our findings show that short-term treatment with ebselen diminishes a laboratory measure of impulsivity and produces a positive bias in emotional processing as measured by the FERT. Based on its ability to inhibit IMPase, ebselen has been proposed as a putative lithium-mimetic, and short-term treatment with ebselen in healthy participants decreased levels of inositol in anterior cingulate cortex, as measured by MRS (Singh et al. 2015). This has been confirmed as part of the present study (Masaki et al. 2016). This is consistent with IMPase inhibition in the brain in humans. At the doses employed and for the short period of time during which treatment was administered, ebselen appeared well tolerated and the only side effect apparently distinguishing it from placebo was drowsiness.

The CGT provides measures of decision-making as well as impulsive responding. The latter (‘delay aversion’) is measured by subtracting the bets made on ascending trials from those of the descending trials. In this task, low bets in the ascend condition coupled with high bets in the descend condition reveal a more impulsive form of responding and this difference in bets on the two conditions was diminished by ebselen suggesting a decrease in impulsivity. Interestingly, patients at risk of bipolar disorder by virtue of a positive family history, or high score on the Hypomanic Personality Scale, demonstrate increased delay aversion on the CGT (Wessa et al. 2015).

The observation that ebselen diminishes impulsivity on the CGT is also of interest in view of the clinical effect of lithium to decrease rates of suicide in patients with recurrent mood disorder (Cipriani et al. 2013). Part of this effect is no doubt due to the action of lithium to diminish the risk of episodes of severe depression. However, lithium appears to have a greater benefit to reduce suicidality than other equally effective mood-stabilizing drugs (Cipriani et al. 2013). In addition, lithium lowers suicidal behaviour even in patients who do not respond well to its mood-stabilizing effects (Ahrens and Müller-Oerlinghausen 2001). This has given rise to the suggestion that part of the reason for lithium’s ability to reduce suicidal behaviour stems from additional neuropsychological actions to inhibit impulsivity and aggression, and work in animal models shows that lithium can indeed produce such effects (O’Donnell and Gould 2007; Ohmura et al. 2012). There are also clinical studies showing that lithium can decrease impulsive aggression in non-mood disorder patient populations (Sheard et al. 1976; Craft et al. 1987; O’Donnell and Gould 2007; Jones et al. 2011).

The CGT also provides a measure of risk taking calculated from the proportion of points bet on the gambles with a greater likelihood of a positive outcome. This has been taken as a measure of reward seeking or loss aversion. Depressed patients and those at risk of depression characteristically have lower reward seeking on the CGT (Murphy et al. 2001; Rawal et al. 2013; Mannie et al. 2015), the opposite of the effect produced by ebselen. This might suggest a potential antidepressant effect of ebselen. To the best of our knowledge, the effect of lithium has not been studied specifically in the CGT but clinically lithium is not usually regarded as a useful acute antidepressant agent when used as monotherapy. However, lithium can have antidepressant effects when added to ineffective antidepressant medication in patients with resistant depression (Nelson et al. 2014). In our previous study, we found evidence for decreased reinforcement learning after ebselen treatment (Singh et al. 2015). The CGT provides a measure of reward responding which is independent of learning (Rogers et al. 1999; Clark et al. 2008). This suggests that the effect of ebselen on reinforcement learning is not mediated by devaluation of reward.

The effects of ebselen on the FERT also suggest a potential antidepressant action. Negative biases in emotional processing are well characterized in depression (Disner et al. 2011), and conventional antidepressants have been shown to produce a positive shift in emotional processing in healthy participants as measured by the FERT (Harmer et al. 2008, 2009; Arnone et al. 2009). A similar effect has been described with novel antidepressants including agomelatine and a nociceptin receptor antagonist (Harmer et al. 2011; Post et al. 2015).

In our previous study of ebselen, we found an increased accuracy of recognition of happy facial expressions but also disgust (Singh et al. 2015) which was not apparent in the present investigation. We are uncertain of the reason for this, but it may reflect differences in experimental design. In our earlier study, the effect of ebselen on the FERT was studied using a parallel group, placebo-controlled design rather than the cross-over design used here, which raises a potential influence of order effects. Also the dose of ebselen in the present study was twice that used previously (Singh et al. 2015). In the present study, we found no main effect of order on the accuracy of recognition of positive and negative faces, though a previous investigation of the FERT did report an increased ability to discriminate expressions of happiness and disgust following repeat testing after an interval of 1 week (Adams et al. 2015). However, we found no interaction between order, emotion and treatment in the present study. Also because the order of ebselen and placebo administration was randomized, we think it is unlikely that order/learning effects played a significant role in the ability of ebselen to increase the recognition of positive facial expressions in the FERT.

In conclusion, ebselen is a potential lithium-mimetic which appears to decrease impulsivity on the CGT. This effect is of particular interest in view of the action of lithium to decrease impulsivity in animal studies and impulsive aggression and suicide in patient populations.

## Electronic supplementary material

Below is the link to the electronic supplementary material.

ESM 1 (DOCX 221 kb)
