# Supplementary material for: Effects of the potential lithium-mimetic, ebselen, on impulsivity and emotional processing
Source: Psychopharmacology (Berl). 2016 Jun 2;233:2655–61. doi: 10.1007/s00213-016-4319-5 (PMC4917572; doi:10.1007/s00213-016-4319-5)
Supplement: Supplementary file 1 — (DOCX 221 kb) [file 213_2016_4319_MOESM1_ESM.docx]

Supplementary Information

**Effects of the potential lithium-mimetic, ebselen, on impulsivity and emotional processing**

Table S1: Baseline personality and mood scores (mean ± SEM)

| EPQ: N | 4.3 ± 0.7 |
| --- | --- |
| EPQ: P | 3.4 ± 0.5 |
| EPQ: L | 6.2 ± 0.7 |
| EPQ: E | 15.3 ± 0.8 |
| BDI | 2.0 ± 0.7 |
| STAI | 27.2 ± 1.2 |

EPQ: Eysenck Personality Questionnaire; BDI: Beck’s Depression Inventory, STAI: state measure of the State-Trait Anxiety Inventory

**Table S2:** Accuracy of recognition of facial expressions of emotion

|  | Placebo  Mean ± SEM | Ebselen  Mean ± SEM | Statistical significance  (paired samples t-test) |
| --- | --- | --- | --- |
| Anger | 20.7 ± 1.3 | 20.85 ± 1.0 | p = 0.836 |
| Disgust | 21.2 ± 1.5 | 21.3 ± 1.3 | p = 0.907 |
| Fear | 22.7 ± 1.231 | 22.15 ± 1.0 | p = 0.520 |
| Happy | 25.0 ± 1.0 | 26.6 ± 0.9 | p = 0.033 |
| Sad | 23.2 ± 1.5 | 21.5 ± 1.3 | p = 0.152 |
| Surprise | 22.9 ± 1.1 | 24.2 ± 1.0 | p = 0.210 |

**
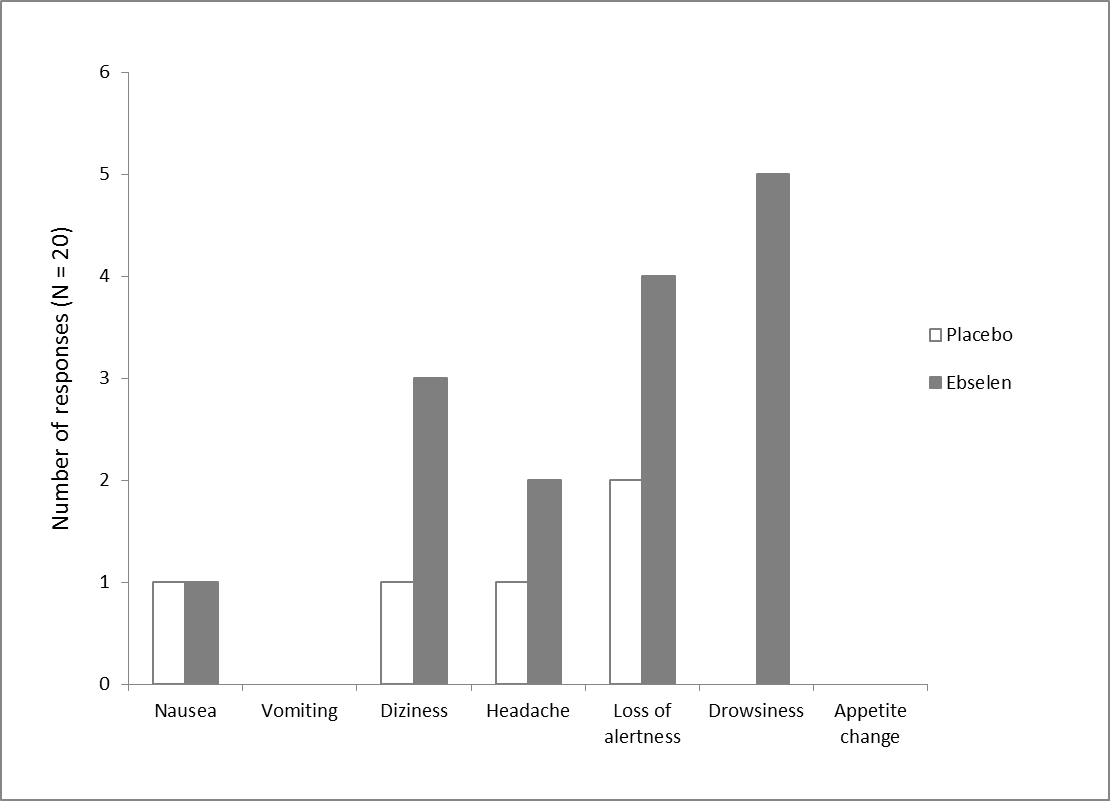
**

**Figure S1:** Comparison of the frequency of side effects reported by participants following placebo (light bars) or ebselen (dark bars) treatment.

**
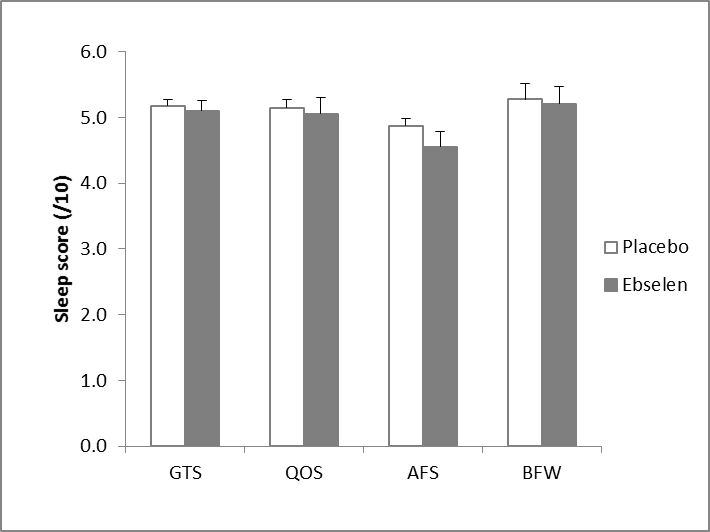
**

**Figure S2:** Sleep assessment following placebo or ebselen treatment. Subjective measures of sleep were taken using the Leeds Sleep Evaluation Questionnaire (LSEQ) on the morning following the second ebselen dose (morning preceding psychological testing). The following LSEQ measures were assessed using a 10 point visual analogue scale: GTS – getting to sleep, QOS – quality of sleep, AFS – awake following sleep, BFW – behaviour following wakening. There were no main effects of treatment (F _1, 19_ = 0.58, p = 0.46) or significant treatment x sleep-measures interactions (F _3, 57_ = 3.90, p = 0.76 on the ANOVA. Values represent mean (out of 10) ± standard error of mean.

**Figure S3:** Effect of ebselen on accuracy of facial expression recognition. Values represent the mean percentage correct for each of the six basic emotions summed over different intensity levels. There was a tendency towards significant interactions between treatment and emotion (F _5, 90_ = 2.15, p = 0.067). Follow-up pairwise comparisons revealed that ebselen treatment was associated with significantly improved accuracy in the recognition of happy facial expressions (* p = 0.033), without significant effects in the recognition of other expressions. All data are plotted as mean ± standard error of mean, N=20.
